# Research progress on molecular mechanisms and signaling pathways of toxic metal exposure-induced lung fibrosis

**DOI:** 10.3389/fimmu.2026.1961991

**Published:** 2026-09-28

**Authors:** Dongmei Zhang, Wenwen Liu, Yanli Yang

**Affiliations:** Respiratory Department, First Hospital of Jilin University, Changchun, China

**Keywords:** autophagy, epigenetic regulation, inflammation, oxidative stress, pulmonary fibrosis, TGF-β/smad signaling, therapeutic strategies, toxic metals

## Abstract

Toxic metal exposure is a pressing global health concern, with accumulating evidence linking it to the onset and progression of pulmonary fibrosis. Mechanistically, metal-induced pulmonary fibrosis operates through a complex, interconnected signaling network rather than a single linear cascade. The initiating role of oxidative stress and the central hub function of the transforming growth factor-β/Smad (TGF-β/Smad) pathway are well-established, whereas the contributions of non-canonical mitogen-activated protein kinase (MAPK), phosphatidylinositol 3-kinase/protein kinase B (PI3K/Akt), Janus kinase/signal transducer and activator of transcription (JAK/STAT), and mammalian target of rapamycin complex 1 (mTORC1) pathways are supported by varying degrees of preclinical evidence, with some crosstalk mechanisms remaining largely hypothetical. Concurrently, persistent Toll-like receptor 4/nuclear factor kappa-B/NOD-like receptor family pyrin domain containing 3 (TLR4/NF-κB/NLRP3)-driven inflammation, macrophage polarization imbalance, impaired autophagy-lysosomal function, and non-apoptotic cell death pathways including ferroptosis and pyroptosis collectively promote alveolar epithelial injury, fibroblast-to-myofibroblast transition, and excessive extracellular matrix deposition. We summarize current therapeutic strategies—ranging from antioxidant/anti-inflammatory agents and TGF-β/autophagy modulators to metal chelators and emerging stem cell/nanomedicine approaches—and highlight their preclinical promise and translational challenges. Major unresolved issues include the translational gap between animal models and real-world human exposure scenarios, controversies over mixed-metal dose-response thresholds, and insufficient understanding of feedback and compensatory mechanisms within the molecular network. We outline future research directions aimed at bridging these gaps, with the goal of establishing a comprehensive theoretical framework for early warning, precision diagnosis, and targeted intervention in toxic metal-related pulmonary fibrosis.

## 1 Introduction

Exposure to toxic metals represents a critical environmental and occupational health challenge worldwide. Environmental and occupational contact with metals such as hexavalent chromium, nickel, cadmium, lead, mercury, and arsenic poses a serious threat to human health (1). With accelerating industrialization and worsening environmental pollution, human exposure occurs through multiple routes, including occupational settings, environmental media, and lifestyle factors. Moreover, because toxic metals are non-biodegradable, they persist and accumulate in the environment, with concentrations steadily rising (2). Toxic metals carried by fine particulate matter can deposit in and dissolve within the lungs, posing significant non-carcinogenic and potential carcinogenic risks (3). Toxic metals cause cellular damage through oxidative stress, DNA repair disruption, and impaired antioxidant defenses. They are also extensively implicated in cardiovascular disease, neurodegeneration, and cancer (2, 4, 5).

Pulmonary fibrosis is a chronic, progressive disease with high incidence, prevalence, and mortality worldwide, characterized by excessive extracellular matrix accumulation in the lung parenchyma (6–8). This condition leads to progressive scarring and irreversible decline in lung function. Pathogenesis involves complex interactions among epithelial, endothelial, immune, and fibroblast cells, alongside dysregulated signaling pathways that control myofibroblast activation, ECM cross-linking, metabolism, and inflammation (9). Recently, a growing body of epidemiological and experimental evidence has identified toxic metal exposure as a considerable environmental risk factor that triggers or accelerates pulmonary fibrosis progression. The relationship between occupational/environmental metal exposure and interstitial lung disease has attracted widespread attention; low-level metal exposure may cause lung damage through both specific immune responses and nonspecific innate immune mechanisms (10). Disruptions in metal ion homeostasis and toxic metal exposure play a pivotal role in the initiation and progression of pulmonary fibrosis (8, 11).

Although several reviews have addressed metal toxicity or pulmonary fibrosis separately, few have systematically integrated multi-metal epidemiological evidence with the intricate, network-level signaling mechanisms specific to metal-induced pulmonary fibrosis. Unlike previous reviews that often focus on a single metal or a linear pathway, this review systematically synthesizes epidemiological, mechanistic, and therapeutic evidence across multiple toxic metals and interconnected signaling networks, with particular emphasis on pathway crosstalk, positive feedback loops, and the translational gaps between preclinical models and real-world human exposure. In doing so, we aim not only to elucidate the complex pathological networks underlying this disease but also to provide a comprehensive theoretical framework for early warning, precision diagnosis, and targeted therapeutic intervention. This review systematically examines the epidemiological associations between toxic metal exposure and pulmonary fibrosis, the underlying cytological and molecular mechanisms, and current therapeutic strategies, with the goal of offering robust scientific support for future research and clinical management.

## 2 Epidemiological evidence and dose-response characteristics

Pulmonary fibrosis incidence has been rising globally, a trend that cannot be fully explained by population aging alone, suggesting a potential role for environmental and occupational factors. However, it is critical to emphasize that the epidemiological evidence reviewed herein primarily establishes statistical associations rather than causal relationships. While observational studies consistently demonstrate positive associations between metal exposure and pulmonary fibrosis or lung function decline, these findings are subject to residual confounding and reverse causality, and causal inference requires corroboration from mechanistic, dose-response, and intervention studies. The association between occupational exposure to metal dust and pulmonary fibrosis risk is supported by extensive epidemiological evidence. Early observational studies in specific U.S. regions revealed significantly elevated fibrosis prevalence in certain “hotspots,” attributed to combined occupational/environmental metal exposure and racial susceptibility (10). Similarly, the increasing number of registered IPF cases in the Silesian Voivodeship of Poland has been linked to occupational metal dust exposure (12). A case series analysis further demonstrated that 41.9% (13/31) of IPF patients and 55.6% (10/18) of patients with unclassified fibrosis had a history of occupational metal dust exposure (13). Park et al. conducted a meta-analysis of 12 case-control studies and found that exposure to metal dust in occupational or environmental settings was associated with a significant increase in IPF risk (OR = 1.83, 95% CI: 1.15–2.91) (14). A systematic review by Pauchet et al. further confirmed that metal exposure increases the risk of IPF by 42% (OR = 1.42, 95% CI: 1.05–1.92) (15).

Beyond occupational settings, population-based studies have further quantified risks associated with specific metals. Each 1-unit increase in urinary copper concentration was significantly associated with elevated pulmonary fibrosis risk (16). In a cross-sectional study of long-term residents in Kaohsiung, China, individuals with blood lead levels in the upper quartile (>1.61–2.30 μg/dL) and the highest quartile (>2.31 μg/dL) exhibited a 33% (OR = 1.33, 95% CI: 1.01–1.75) and 36% (OR = 1.36, 95% CI: 1.01–1.82) increased risk, respectively, with a significant dose-response trend (P = 0.030) (17). Arsenic exposure has received particular attention in longitudinal investigations. A cohort study near a petrochemical industrial park confirmed that individuals with urinary arsenic levels in the top sixth percentile (>209.7 μg/g creatinine) had increased fibrosis risk at baseline (OR = 1.87, 95% CI: 1.16–3.02) and follow-up (OR = 1.74, 95% CI: 1.06–2.84) (18). These findings represent observational associations and do not establish causality, as unmeasured confounding and exposure misclassification cannot be entirely excluded.

Epidemiological and cohort studies also consistently demonstrate significant negative correlations between metal exposure and key lung function indicators, reflecting the physiological impact of metal-induced structural damage. Among welders, lead and cadmium were significantly and negatively correlated with forced vital capacity (FVC) and forced expiratory volume in 1 second (FEV_1_), while nickel and chromium were negatively correlated with peak expiratory flow (PEF) (19). Mixed-metal effect analyses indicate that combined exposure to lead, cadmium, nickel, and chromium has a significant overall negative impact on lung function (19, 20). In the general population, serum cadmium and lead levels are negatively correlated with the FEV_1_/FVC ratio (21, 22). A cohort study of women revealed that, among premenopausal women, cadmium and lead were negatively correlated with FEV_1_/FVC; among postmenopausal women, combined cadmium and mercury exposure also reduced this ratio (22). In rural northwestern China, elevated urinary antimony, mercury, and manganese, as well as reduced molybdenum, were associated with impaired pulmonary function (23). Toxic transition metals in fine particulate matter (PM) have been identified as major contributors to pulmonary toxicity, significantly associated with pro-inflammatory cytokines and lung injury biomarkers (24). Long-term metal exposure data further identify tungsten as a novel risk factor for chronic obstructive pulmonary disease (COPD) (25). The association between heavy metal exposure and COPD has been quantitatively confirmed. High blood levels of cadmium and lead are both associated with a significantly increased risk of COPD (26, 27).

Importantly, the observed associations are markedly influenced by population heterogeneity and confounding factors, which require careful interpretation. Smoking represents the most prominent confounder, as it is independently associated with both altered metal toxicokinetics (e.g., cadmium accumulation) and pulmonary fibrosis risk. While most included studies adjusted for smoking status, residual confounding from pack-year history or secondhand smoke exposure cannot be fully excluded. Occupational co-exposures—such as concurrent silica, asbestos, or organic dusts—frequently accompany metal exposure in industrial settings, and few studies have fully disentangled their independent effects. Age is another critical modifier, as elderly individuals exhibit reduced renal clearance and greater cumulative exposure burden, while sex differences, particularly menopausal status in women, may influence metal distribution and susceptibility due to hormonal regulation of metal transporters. Additionally, socioeconomic status and nutritional factors (e.g., iron, zinc, or selenium status) can modify both metal absorption and antioxidant defense capacity, yet these variables are often underreported. Several studies have revealed notable susceptibility differences across subgroups, including age- and sex-specific effects, as well as preliminary evidence for racial disparities. Furthermore, while most observational studies adjust for a core set of confounders, residual confounding from unmeasured or imprecisely measured variables—such as dietary patterns, occupational history duration, and genetic polymorphisms in metal-metabolizing enzymes—remains a persistent challenge. Thus, the epidemiological evidence should be interpreted as supportive of an association that is biologically plausible and mechanistically supported, rather than definitive proof of causation. Future prospective cohorts with repeated exposure measurements, detailed covariate collection, and Mendelian randomization designs are needed to strengthen causal inference.

Beyond single-metal effects, emerging evidence points to complex nonlinear dose-response relationships and metal-metal interactions that challenge the definition of safe exposure thresholds. For instance, mercury exhibits an inverted U-shaped relationship with FEV_1_/FVC (22), while lead, cadmium, and manganese show threshold effects and nonlinear associations with lung function decline (21). Cadmium also displays a nonlinear relationship with FVC. At the mixture level, interactions further complicate risk assessment—arsenic exhibits negative interactions with other metals in renal injury models, whereas manganese shows positive interactions with nickel (93). *In vitro* experiments confirm that cadmium and copper exhibit the strongest synergistic effects, whereas iron and zinc exhibit the strongest antagonistic effects (28). These nonlinearities and interaction effects represent a critical area of ongoing controversy, as most current studies fail to report clear dose-response thresholds, resulting in a lack of precise quantitative evidence to inform environmental standards and clinical risk assessments (21, 29). This translational and methodological gap will be further critically examined in Section 5, where we discuss the limitations of existing models and the unresolved threshold controversies.

## 3 Core signaling pathways and molecular mechanisms of toxic metal-induced pulmonary fibrosis

The process by which metal exposure induces pulmonary fibrosis involves a highly complex network of molecules and signaling cascades. Following environmental or occupational exposure to toxic metals such as cadmium, lead, and nickel, the pulmonary microenvironment undergoes significant changes, triggering oxidative stress, inflammatory responses, and excessive activation of fibrogenic pathways. These core signaling pathways not only function independently but also interact extensively with one another, collectively driving alveolar epithelial cell damage, fibroblast activation, and extracellular matrix deposition. A thorough understanding of these molecular mechanisms holds significant translational clinical value for identifying early intervention targets and developing anti-fibrotic drugs. To facilitate cross-metal comparison and highlight the distinction between metal-specific and shared fibrotic responses, we summarize the key experimental evidence, affected cell types, major signaling pathways, and relative evidence strength for each metal in **Table 1**. This comparative framework is intended to help readers distinguish mechanisms that are consistently observed across metals from those that appear unique to metal types or experimental contexts.

**Table 1:** Comparative summary of metal-specific and common mechanisms in toxic metal-induced pulmonary fibrosis.

Metal	Key experimental models	Primary target cells	Major Signaling pathways involved	Metal-specific features	Shared/common mechanisms	Evidence strength*
Cadmium (Cd)	Mouse intratracheal instillation; duck model; HEL299 fibroblasts	Alveolar epithelial cells; fibroblasts	TGF-β/Smad; Nrf2/HO-1 (inhibition); AKT/PI3K; mTORC1; MAPK/ERK	Ferroptosis via GAPDH aggregation; Nrf2/HO-1 inhibition; SIRT6 downregulation	ROS overproduction; TGF-β/Smad activation; NLRP3 inflammasome; autophagy dysregulation	*** (well-established)
Nickel (Ni)	Mouse inhalation; NiO nanoparticle models	Alveolar epithelial cells; fibroblasts	TGF-β/Smad; JAK2/STAT3; MAPK/ERK; Hedgehog/autophagy	JAK2/STAT3 synergistic amplification; Hedgehog pathway-mediated autophagy suppression	Oxidative stress; EMT; collagen deposition; ECM remodeling	*** (well-established for TGF-β/Smad; ** for JAK2/STAT3)
Arsenic (As)	*In vitro* cell culture; epidemiological cohorts	Bronchial epithelial cells	TGF-β/Smad (direct receptor binding); MAPK/ERK	Direct TGF-β receptor binding; bidirectional regulation of Smad signaling	Epithelial injury; inflammatory cytokine release	*** (well-established for receptor binding)
Chromium (Cr, hexavalent)	*In vitro* epithelial cell models	Pulmonary epithelial cells	MAPK (strong activation); oxidative stress pathways	Strong MAPK activation; acute lung injury profile	ROS production; DNA damage; inflammatory response	** (moderate, mostly *in vitro*)
Copper (Cu)	*In vitro* epithelial cells; population-based studies	Alveolar epithelial cells	MAPK (MMP-3 upregulation); TGF-β/Smad; autophagy	MMP-3-mediated EMT; copper transporter (CTR1) involvement; lysyl oxidase (LOX) pathway	Mitochondrial ROS; epithelial-mesenchymal transition	** (moderate, emerging)
Lead (Pb)	Lung fibroblast culture; mouse models	Fibroblasts; alveolar epithelial cells	MAPK/ERK (paracrine activation)	Paracrine ERK activation in epithelial cells via fibroblast-secreted factors	Fibroblast activation; apoptosis; ECM production	** (moderate, limited mechanistic detail)
Manganese (Mn)	*In vitro* fibroblast/epithelial co-culture	Fibroblasts; alveolar epithelial cells	MAPK/ERK	ERK pathway activation	ROS production; fibrosis marker upregulation	* (limited, preliminary)
Molybdenum (Mo)	Duck model (co-exposure with Cd)	Macrophages; pulmonary tissue	TGF-β/Smad (in combination with Cd); oxidative stress	Synergistic effects with Cd; M1 macrophage polarization	Inflammation; oxidative stress; ECM deposition	* (limited, single species)

*Evidence strength rating: ***well-established with multiple models and consistent findings; **moderate, supported by select studies but with gaps; *limited, preliminary evidence requiring further validation. Shared mechanisms include ROS overproduction, TGF-β/Smad activation, NLRP3 inflammasome, autophagy dysregulation, ferroptosis, and pyroptosis, though the relative contribution of each varies across metals.

### 3.1 Oxidative stress and ROS-mediated damage cascades

Oxidative stress has been consistently shown to be a key mechanism in the onset and progression of toxic metal-induced pulmonary fibrosis (30, 31) (**Figure 1**). Exposure to toxic metals can penetrate deep into the lungs, disrupting the redox balance of lung tissue and leading to excessive production of ROS, such as superoxide anions, hydroxyl radicals, and hydrogen peroxide (31, 32). This is specifically manifested by elevated levels of hydrogen peroxide and malondialdehyde in lung tissue, accompanied by a significant decrease in the activities of antioxidant enzymes, including superoxide dismutase, glutathione peroxidase, and catalase (33, 34). Cadmium exposure can inhibit the Nrf2/HO-1 signaling pathway, leading to increased ROS levels and lipid peroxidation, which in turn accelerates endoplasmic reticulum stress and promotes ferroptosis (35). In a cadmium-induced pulmonary fibrosis model, the aggregation of glyceraldehyde-3-phosphate isomerase 1 leads to impaired glycolysis, accompanied by mitochondrial damage, iron deposition, and increased lipid peroxidation, which further drive ferroptosis and the fibrosis process (11). Crucially, ferroptosis is intrinsically linked to ROS overproduction—iron-dependent lipid peroxidation generates massive ROS, which in turn perpetuates mitochondrial dysfunction and creates a vicious cycle of oxidative injury that directly promotes fibroblast activation and ECM deposition. The molecular machinery and upstream triggers of ferroptosis, as well as its intersection with pyroptosis and the broader fibrotic network, are discussed in greater detail in Section 3.5. Nickel exposure also leads to elevated malondialdehyde levels, reduced total antioxidant capacity, and decreased superoxide dismutase activity, indicating that oxidative stress plays a key mediating role in nickel-induced pulmonary fibrosis (36). Exposure to sodium chromate can induce significant oxidative stress in pulmonary epithelial cells and strongly activate the MAPK signaling pathway, leading to acute lung injury (37). Exposure to copper oxide nanoparticles can induce an increase in mitochondrial-derived ROS production, which in turn activates the MAPK pathway and upregulates matrix metalloproteinase-3, thereby mediating epithelial-mesenchymal transition (EMT) (38). At the level of signal transduction, 4-hydroxy-2-nonenal (4-HNE), a derivative of polyunsaturated fatty acids, acts as a highly reactive second messenger that sustains inflammatory and fibrotic processes by transmitting the effects of reactive oxygen species (ROS) (6). Importantly, 4-HNE can directly modify Smad3 or upstream TGF-β receptors via covalent adduction, thereby lowering the threshold for TGF-β activation and amplifying downstream fibrotic signaling even in the absence of high ligand concentrations. The NADPH oxidase DUOX1 is induced following lung injury; the hydrogen peroxide it produces inhibits the interaction between phosphorylated Smad3 and the ubiquitin ligase NEDD4L, thereby preventing the ubiquitination and degradation of p-Smad3. This prolongs Smad3 phosphorylation, creating a positive feedback loop that amplifies TGF-β signaling (32). Interventions targeting oxidative stress show anti-fibrotic potential. Activation of Nrf2 or antioxidant supplementation reduces ROS levels and alleviates fibrotic pathology. Mitochondria-targeted coenzyme Q significantly reduces mitochondrial ROS and restores redox balance.

**Figure 1:**
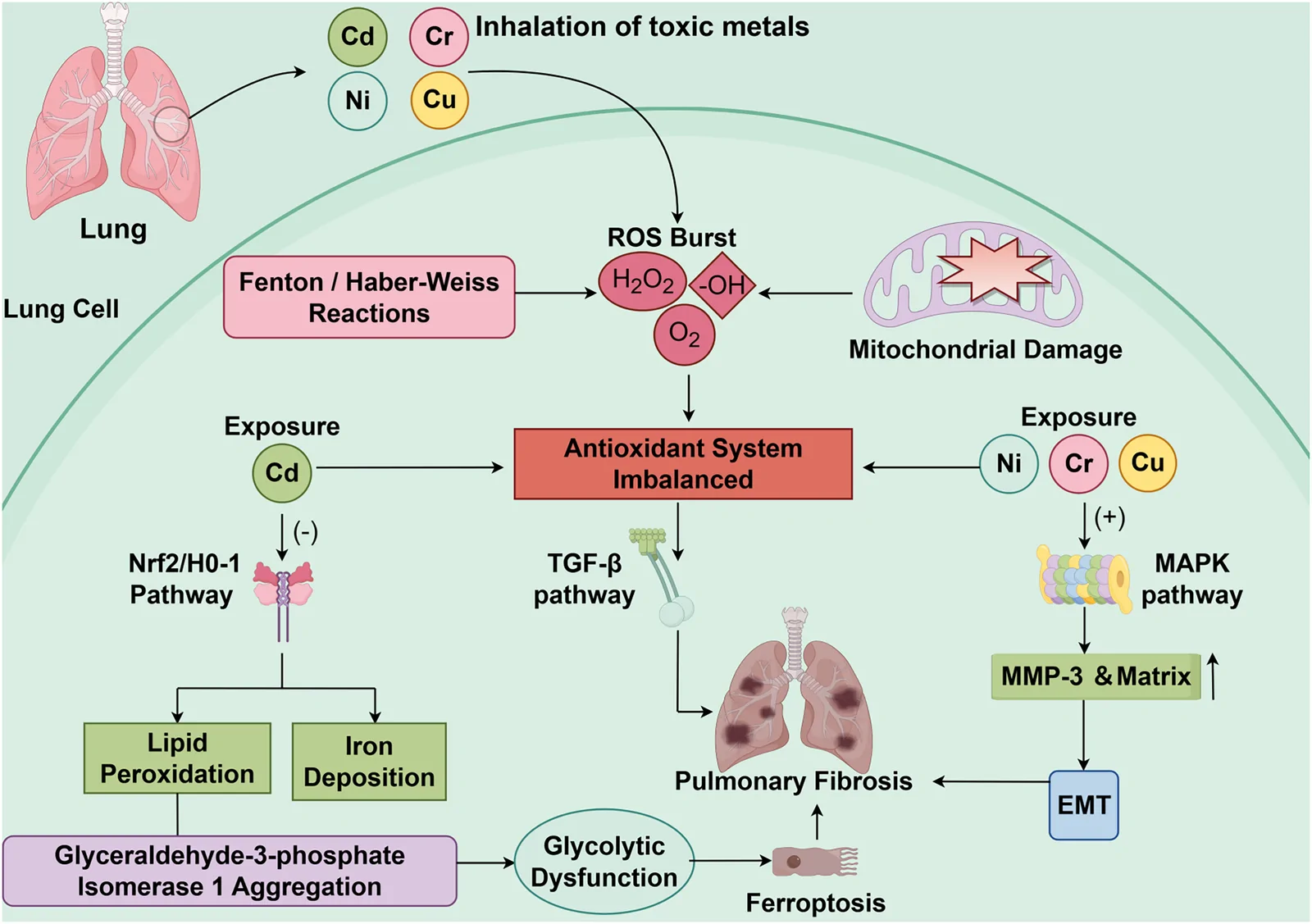
Schematic diagram of oxidative stress, ferroptosis, and downstream signaling cascades in toxic metal-induced pulmonary fibrosis. This figure illustrates the cascade of oxidative injury and fibrotic signaling triggered by toxic metal exposure to the lung. Toxic metals (cadmium, nickel, chromium, and copper) penetrate lung tissue and induce a reactive oxygen species (ROS) burst, characterized by excessive production of H_2_O_2_, hydroxyl radicals (·OH), and superoxide anions (O_2_·^-^). The resulting oxidative stress causes mitochondrial damage and disrupts the antioxidant system, notably through inhibition of the Nrf2/HO-1 pathway (exemplified by cadmium exposure). These events collectively promote lipid peroxidation, iron deposition, and glycolytic dysfunction, which converge to drive ferroptosis—a novel iron-dependent cell death pathway that amplifies fibrotic injury. Simultaneously, ROS overproduction activates the MAPK pathway, upregulates matrix metalloproteinase-3 (MMP-3), and promotes extracellular matrix (ECM) remodeling, while the TGF-β pathway is engaged to drive fibroblast activation. The integrated network involving ferroptosis, MAPK, and TGF-β signaling synergistically promotes epithelial-mesenchymal transition (EMT) and ultimately leads to pulmonary fibrosis. The figure also highlights the interconnection between Nrf2/HO-1 dysregulation and cadmium-induced oxidative damage, identifying this axis as a critical node for therapeutic intervention. Cd, cadmium; Cr, chromium; Cu, copper; ECM, extracellular matrix; EMT, epithelial-mesenchymal transition; MAPK, mitogen-activated protein kinase; MMP-3, matrix metalloproteinase-3; Ni, nickel; Nrf2, nuclear factor erythroid 2-related factor 2; ROS, reactive oxygen species; TGF-β, transforming growth factor-beta.

### 3.2 TGF-β/smad signaling network

The TGF-β signaling pathway serves as the central regulatory hub driving pulmonary fibrosis, encompassing both Smad-dependent classical and non-classical pathways such as MAPK, PI3K/Akt, JAK/STAT, and ROCK (39–42) (**Figure 2**). The canonical Smad-dependent arm is unequivocally established in metal-induced fibrosis, supported by consistent findings across multiple metal types and experimental models. In contrast, the non-canonical branches are supported by more heterogeneous evidence—some are well-documented for specific metals (e.g., cadmium-induced AKT/PI3K activation), while others derive from single studies or extrapolation from non-metal fibrosis models and should therefore be interpreted with appropriate caution. Toxic metals exploit this hub through multiple mechanisms, and the pathway’s complexity warrants a hierarchical examination.

**Figure 2:**
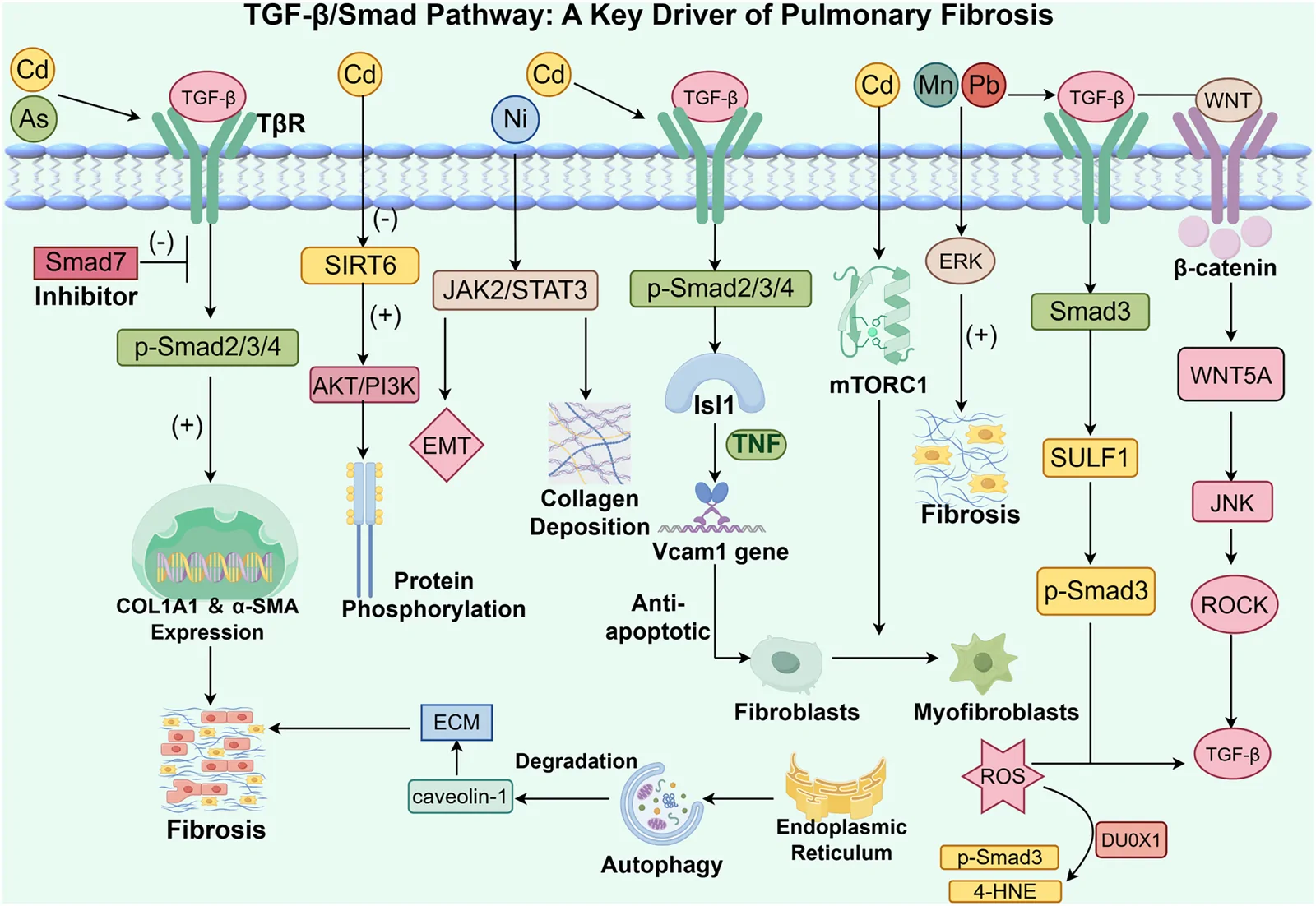
The TGF-β/Smad signaling network as the central hub in metal-induced fibrosis. This image depicts the hierarchical organization of the TGF-β signaling network. Toxic metals (cadmium, arsenic, nickel) directly bind to TGF-β receptors, activating the canonical Smad2/3 pathway and suppressing the inhibitory Smad7. Concurrently, non-canonical pathways—including MAPK/ERK, PI3K/AKT, JAK/STAT, and mTORC1—are engaged, synergistically promoting epithelial-mesenchymal transition (EMT), collagen synthesis, and myofibroblast differentiation. The schematic highlights critical crosstalk mechanisms: 4-HNE-mediated receptor sensitization, WNT5A/JNK/ROCK axis activation of latent TGF-β, and SULF1-driven positive feedback. This integrated network transforms acute injury into self-perpetuating fibrotic progression. EMT, epithelial-mesenchymal transition; ECM, extracellular matrix; ROCK, Rho-associated protein kinase.

#### 3.2.1 Canonical smad-dependent signaling

Toxic metals such as cadmium and arsenic can bind directly to the TGF-β receptor, inducing conformational changes and promoting phosphorylation, thereby activating the classical Smad signaling cascade (33, 43). Combined exposure to molybdenum and cadmium induces fibrosis via the TGF-β1/Smad2/3 pathway, evidenced by increased COL1A1 and α-SMA expression and decreased expression of the inhibitory Smad7 (33, 44). Nickel exposure not only activates the TGF-β1/Smad pathway but also upregulates JAK2/STAT3 signaling, synergistically promoting EMT and collagen deposition (36, 45). TGF-β/Smad3 can indirectly regulate the TNF pathway gene Vcam1 via the transcription factor Isl1, driving fibroblast resistance to apoptosis and coordinating genome-wide alternative splicing events to promote fibrosis progression (46, 47). Critically, Smad7 serves as the primary endogenous brake on this pathway; its downregulation by toxic metals removes a key inhibitory checkpoint, and therapeutic strategies aimed at restoring Smad7 expression or function represent a promising but underexplored direction for intervention. However, direct evidence for Smad7 restoration in metal-specific fibrosis models remains limited, and this approach currently rests largely on extrapolation from TGF-driven fibrotic paradigms rather than metal-exposure-specific validation.

#### 3.2.2 Non-canonical pathways

Non-canonical pathways are deeply involved in toxic metal-induced lung toxicity. Cadmium exposure suppresses SIRT6 expression, enhances AKT/PI3K phosphorylation, promotes vimentin phosphorylation, and increases extracellular vimentin levels, contributing to fibrosis (48). Low-dose cadmium also activates the mTORC1 pathway, increasing ribosomal protein S6 phosphorylation and providing metabolic support for fibroblast-to-myofibroblast transdifferentiation (49). Exposure to manganese and lead significantly activates the ERK signaling pathway and upregulates fibrosis markers in pulmonary fibroblasts and alveolar epithelial cells (50). Nickel-induced JAK2/STAT3 activation synergizes with TGF-β/Smad signaling to amplify collagen synthesis (36, 45). These non-canonical pathways not only parallel Smad signaling but also intersect with multiple nodes, enabling signal amplification and diversification of fibrotic outputs.

#### 3.2.3 Pathway crosstalk and positive feedback loops

Beyond parallel activation, extensive crosstalk among pathways creates self-reinforcing fibrotic circuits. The WNT5A/JNK/ROCK axis mediates rapid activation of latent TGF-β, promoting myofibroblast transformation in a synergistic rather than merely additive manner with canonical Smad signaling (46). The TGF-β/Smad and Wnt/β-catenin pathways act synergistically; their combined inhibition completely blocks fibrosis in epithelial cells (51). At the microenvironmental level, SULF1 catalyzes the release of TGF-β1 from its receptor, activating the TGF-β1/Smad pathway and forming a positive feedback loop (52).ER stress-mediated autophagy degrades caveolin-1, lifting its inhibition of the TGF-β1/Smad3 pathway and promoting excessive ECM production (53). These interconnected feedback loops—whereby ROS stabilizes p-Smad3 (via DUOX1), 4-HNE sensitizes receptors, and downstream ECM components further activate TGF-β—are supported by evidence from selected *in vitro* and *in vivo* models. However, the extent to which each loop operates concurrently in chronic, low-dose metal exposure scenarios and whether their relative contributions vary across metal types remain unresolved questions, as most supporting data derive from acute exposure or overexpression systems.

A cross-metal comparison reveals that while the core fibrotic machinery—TGF-β/Smad activation, ROS production, and inflammatory signaling—is consistently engaged across multiple metals, each metal also exhibits distinct mechanistic fingerprints. Cadmium stands out for its unique convergence on ferroptosis and Nrf2/HO-1 inhibition; nickel for its prominent JAK2/STAT3 synergy and Hedgehog-autophagy axis; copper for its MMP-3-driven EMT and lysyl oxidase pathway; and arsenic for its direct receptor binding. Lead and manganese, by contrast, appear to rely more heavily on ERK-dependent paracrine signaling, though the evidence base for these metals is comparatively limited. This heterogeneity underscores the need for metal-specific risk assessment and therapeutic targeting, while also highlighting common nodes—particularly TGF-β/Smad and oxidative stress—that may offer broad-spectrum anti-fibrotic opportunities.

### 3.3 The inflammatory response network and the regulation of fibroblast-stimulating cytokine release

The persistent inflammatory response triggered by toxic metal exposure is a key driver of microenvironmental remodeling in pulmonary fibrosis (**Figure 3**). Toxic metals activate the TLR4/NF-κB/NLRP3 inflammasome pathway, upregulating pro-inflammatory factors such as caspase-1, IL-18, and IL-1β, and disrupting macrophage polarization balance (33, 54, 55). Toxic transition metals in fine particulate matter are significantly associated with IL-6 and TNF-α, as well as lung injury markers (20, 33). PM2.5 drives fibrosis by activating the NF-κB/NLRP3 pathway, and its inhibition significantly reduces TNF-α, IL-1β, and IL-6 levels (55, 56). Exposure to a mixture of toxic metals can upregulate the expression of inflammatory genes, such as IL-8 and TNF, and activate key transcription factors, such as NF-κB (20).

**Figure 3:**
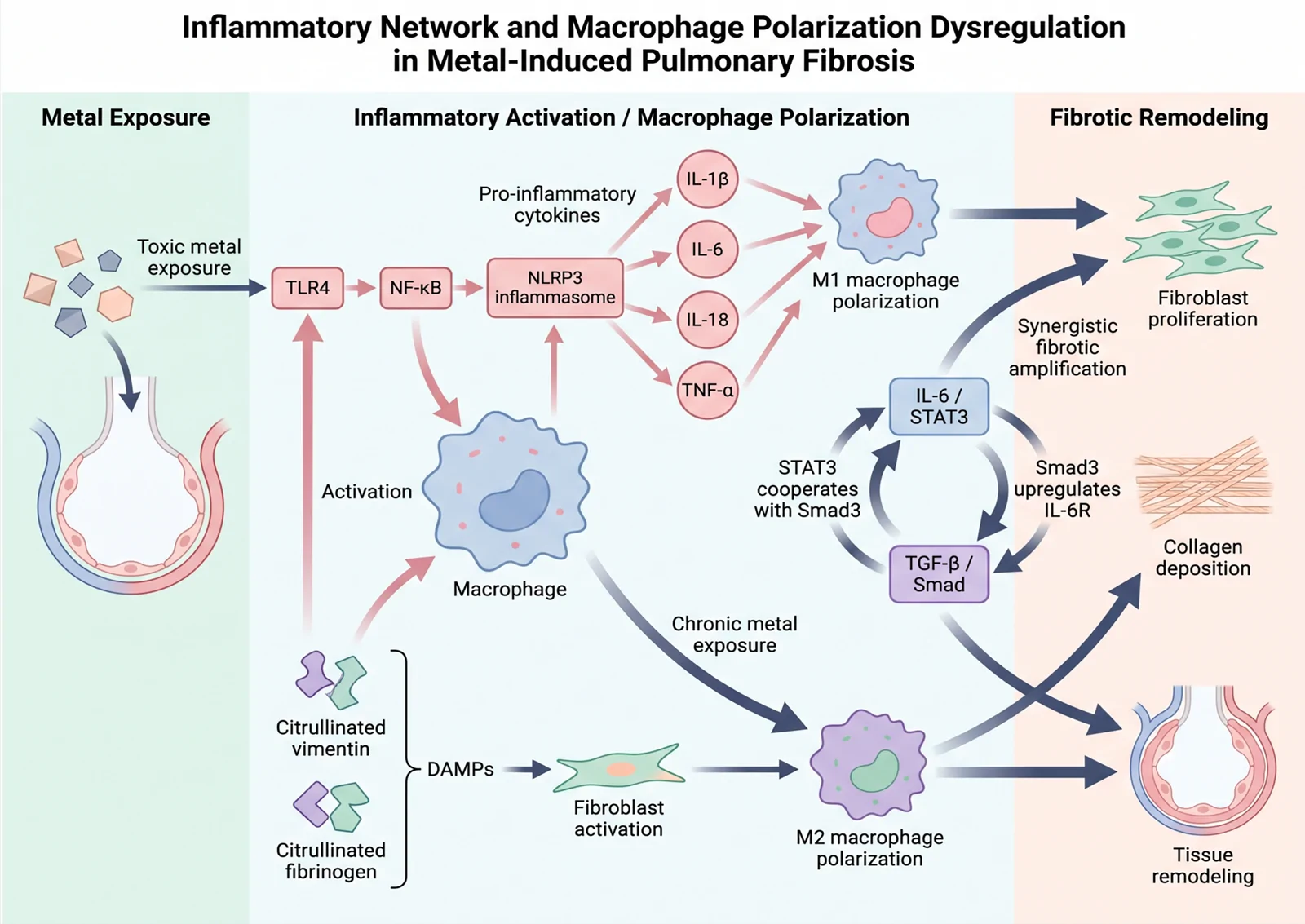
Schematic diagram of the inflammatory network and macrophage polarization dysregulation in metal-induced pulmonary fibrosis. This image illustrates the interconnected inflammatory signaling cascades triggered by toxic metal exposure in the lung microenvironment. Toxic metals activate the TLR4/NF-κB/NLRP3 inflammasome pathway, leading to increased production of pro-inflammatory cytokines (IL-1β, IL-6, IL-18, and TNF-α) and promoting M1 macrophage polarization. Citrullinated vimentin and fibrinogen act as DAMPs that further enhance TLR4-dependent fibroblast activation. Chronic metal exposure drives a shift toward M2 macrophage polarization, which stimulates fibroblast proliferation, collagen deposition, and tissue remodeling. A bidirectional amplification loop between IL-6/STAT3 and TGF-β/Smad signaling is depicted, as STAT3 cooperates with Smad3 on shared target gene promoters while TGF-β-induced Smad3 upregulates IL-6 receptors. JAK/STAT pathway activation synergistically amplifies fibrotic outputs, and pharmacological inhibition of NF-κB or NLRP3 has been shown to reduce TNF-α, IL-1β, and IL-6 release, thereby ameliorating fibrosis-related pathology. DAMP, damage-associated molecular pattern; IL, interleukin; NF-κB, nuclear factor kappa-B; NLRP3, NOD-like receptor family pyrin domain containing 3; STAT3, signal transducer and activator of transcription 3; TGF-β, transforming growth factor-beta; TLR4, Toll-like receptor 4; TNF-α, tumor necrosis factor-alpha.

At the molecular level, in a combined cadmium and carbon black model, citrullinated myosin-1A acts as a DAMP to activate NF-κB in a TLR4-dependent manner, inducing TGF-β1, CTGF, and IL-8 production, and increasing TLR4 expression on pulmonary fibroblasts, thereby promoting fibroblast invasion (57). Cadmium enhances fibrinogen secretion in a CDC2-dependent manner; fibrinogen mediates PAD2-dependent macrophage activation and enhances TLR4-dependent collagen synthesis and cytokine production (58). Combined lead and cadmium exposure upregulates pro-inflammatory cytokines and chemokines, with MMP-12-expressing macrophages driving lung inflammation.

A critical distinction must be made regarding macrophage polarization: while M1 macrophages initiate and sustain acute inflammation, persistent M2 polarization—often driven by chronic metal exposure—actively promotes fibroblast activation, collagen deposition, and tissue remodeling, forming a pro-fibrotic loop that sustains fibrosis even after the initial inflammatory trigger subsides (59). This M2-driven pro-fibrotic paradigm is well-established in idiopathic pulmonary fibrosis and has been observed in select metal-exposure models, but its generalizability across different metals and exposure durations requires further systematic investigation. The IL-6 signaling pathway plays a significant role in immune microenvironment remodeling. Inhibition of IL-6 blocks TGF-β1-induced fibroblast-to-myofibroblast conversion via the STAT3 pathway (60). Notably, IL-6/STAT3 signaling intersects with TGF-β/Smad at multiple levels: STAT3 can directly cooperate with Smad3 on shared target gene promoters, while TGF-β-induced Smad3 upregulates IL-6 receptors, establishing a bidirectional amplification loop between inflammation and fibrosis. The JAK/STAT pathway is also involved; inhibition of the JAK1/STAT3/SOCS3/IL-17A axis alleviates experimental pulmonary fibrosis (61). Targeted modulation of TGF-β/Smad signaling can interfere with macrophage phenotypic conversion and inflammatory factor secretion (59). Intervention strategies targeting the inflammatory network—such as inhibiting NF-κB or downregulating NLRP3—significantly reduce TNF-α, IL-1β, and IL-6 release, improving fibrosis-related pathology (60, 62).

### 3.4 The role of impaired autophagy and lysosomal dysfunction in the progression of fibrosis

The autophagy-lysosomal pathway is centrally involved in maintaining cellular homeostasis and clearing damaged organelles. Exposure to toxic metals significantly disrupts this process, and both insufficient and excessive autophagy can promote epithelial-mesenchymal transition and abnormal epithelial-fibroblast crosstalk (63). In a nickel oxide nanoparticle model, autophagy activity was significantly suppressed, leading to increased collagen deposition; however, lncRNA MEG3 overexpression restored autophagic flux and reduced collagen formation (64). Environmental particulate matter reduces caveolin-1 levels through ER stress and autophagy pathways, activating TGF-β1/Smad3 signaling (53). Conversely, blocking autophagy increases caveolin-1 and improves fibrosis (53). In certain scenarios, autophagy may be abnormally activated: cadmium increases LC3B and p62 expression, enhancing autophagic cell death, whereas 3,3’-diindolylmethane inhibits this excessive death and protects cells (65). Nanoscale indium tin oxide also induces autophagosome accumulation with apoptotic pathway activation (66). Excessive macrophage autophagy exacerbates fibrosis by promoting fibroblast proliferation and migration (67).

The functional consequences of autophagy in pulmonary fibrosis are strikingly cell-type-specific, and this heterogeneity is critical for understanding its dual role. In alveolar epithelial cells (particularly type II), impaired autophagy promotes senescence, ER stress, and EMT, directly contributing to epithelial barrier dysfunction and fibroblast activation. Conversely, maintaining autophagic flux in epithelial cells limits mitochondrial ROS and prevents NLRP3 inflammasome hyperactivation. In fibroblasts, autophagy generally serves a protective role; its inhibition promotes myofibroblast differentiation and excessive ECM production, while its activation limits collagen deposition. In macrophages, the role is context-dependent: basal autophagy restricts NLRP3 inflammasome activation and M1 polarization, but excessive autophagy may promote the release of pro-fibrotic mediators that stimulate fibroblast activity. This cell-type specificity implies that global modulation of autophagy may produce opposing outcomes depending on the dominant cell type affected, highlighting the need for cell-targeted therapeutic strategies. It should be noted that the cell-type-specific model of autophagy in metal-induced fibrosis is largely derived from pharmacological inhibition studies and a limited number of genetic models, and its direct validation in metal-exposure systems remains incomplete.

At the molecular regulatory level, the PI3K/AKT/mTOR pathway is a central autophagy regulator; inhibiting this pathway activates autophagy and alleviates fibrosis (63, 68). Autophagy also intertwines with oxidative stress and inflammasome activation: autophagy inhibition leads to ROS accumulation and excessive NLRP3 activation, whereas autophagy activation suppresses oxidative stress and NLRP3 (69, 70). Additionally, autophagy alleviates fibrosis by activating the Keap1/Nrf2 pathway and maintaining redox homeostasis (69, 71). In a cadmium exposure model, cadmium-induced ER stress is closely associated with impaired autophagy; Nrf2/HO-1 activation suppresses PERK-eIF2α-ATF4-mediated ER stress, improves mitochondrial morphology, and alleviates lung injury (35). Targeting autophagy-related proteins or modulating autophagy has thus become an important strategy for intervening in toxic metal-induced pulmonary toxicity.

### 3.5 Emerging roles of ferroptosis and pyroptosis in metal-induced pulmonary fibrosis

Beyond classical apoptosis and autophagy, recent evidence has identified ferroptosis and pyroptosis as two novel cell death modalities critically involved in metal-induced pulmonary fibrosis. Unlike apoptosis, which is typically non-inflammatory and caspase-dependent, both ferroptosis and pyroptosis are inherently pro-inflammatory and can perpetuate fibrotic remodeling through the release of damage-associated molecular patterns (DAMPs) that activate neighboring macrophages and fibroblasts. A growing body of evidence implicates these pathways in alveolar epithelial type II (AECII) cell injury—a key initiating event in pulmonary fibrosis—and positions them as potential upstream triggers of the inflammatory and fibrotic cascades discussed in previous sections.

#### 3.5.1 Ferroptosis: iron-dependent lipid peroxidation driving epithelial injury and fibroblast activation

Ferroptosis is a non-apoptotic, iron-dependent form of regulated cell death characterized by lethal accumulation of lipid peroxides, glutathione depletion, and inactivation of glutathione peroxidase 4 (GPX4). Toxic metals—particularly cadmium, iron, and chromium—have been shown to induce ferroptosis in lung epithelial cells through multiple convergent mechanisms. Cadmium exposure inhibits the Nrf2/HO-1 antioxidant axis, depletes intracellular glutathione, and promotes iron accumulation and lipid peroxidation, culminating in ferroptotic cell death (35). Mechanistically, cadmium induces aggregation of glyceraldehyde-3-phosphate isomerase 1 (GAPDH), which impairs glycolysis, disrupts mitochondrial function, and exacerbates iron-dependent oxidative injury, forming a vicious cycle that amplifies fibrotic signaling (11). The ferroptosis inhibitor Liproxstatin-1 effectively attenuates these changes, reducing inflammatory and fibrotic gene expression in cadmium-exposed models (11). Beyond cadmium, hexavalent chromium and iron overload conditions have also been linked to ferroptotic epithelial injury, although the mechanistic details remain less well characterized.

Critically, ferroptotic AECII cells release pro-inflammatory DAMPs—including oxidized lipids, high-mobility group box 1 (HMGB1), and mitochondrial DNA—that activate adjacent macrophages via TLR4 and NLRP3 inflammasome pathways. This DAMP-mediated crosstalk establishes a feed-forward loop: ferroptosis begets inflammation, which in turn promotes fibroblast-to-myofibroblast differentiation and extracellular matrix deposition. Consistent with this model, pharmacological inhibition of ferroptosis has been shown to reduce macrophage infiltration and collagen accumulation in preclinical fibrosis models (11, 35). However, whether ferroptosis is a driver or a consequence of the fibrotic microenvironment remains an open question, and the relative contribution of ferroptosis versus apoptosis to AECII depletion in chronic metal exposure scenarios warrants systematic investigation using lineage-tracing and genetic approaches.

#### 3.5.2 Pyroptosis: inflammasome-mediated pore formation and inflammatory amplification

Pyroptosis is a lytic, pro-inflammatory form of cell death executed by the gasdermin family of pore-forming proteins, typically following activation of the NLRP3 inflammasome and caspase-1. In the context of metal-induced lung injury, cadmium and vanadium exposure have been shown to trigger pyroptosis in alveolar epithelial cells through mitochondrial ROS generation and activation of the cGAS-STING pathway (72, 73). Specifically, cadmium induces mitochondrial oxidative stress, which releases mitochondrial DNA into the cytosol, activating cGAS-STING and subsequently promoting NLRP3 inflammasome assembly, caspase-1 cleavage, and gasdermin D (GSDMD)-mediated pore formation (72). This pyroptotic cascade results in the release of IL-1β and IL-18, along with cytosolic DAMPs such as HMGB1 and ATP, which potently activate resident macrophages and recruit circulating inflammatory cells (55, 72).

Notably, pyroptosis intersects with the TGF-β/Smad and inflammatory networks discussed in Sections 3.2 and 3.3. NLRP3-derived IL-1β can directly activate fibroblasts and promote TGF-β1 production from macrophages, establishing a pro-fibrotic amplification loop (55, 62). Moreover, GSDMD-mediated membrane permeabilization may facilitate the release of intracellular metal ions, further amplify oxidative stress, and perpetuate a cycle of injury and inflammation (73). In PM_2.5_-driven pulmonary fibrosis models, inhibition of the NLRP3 pathway significantly attenuates pyroptotic cell death and reduces downstream fibrotic markers (55), suggesting that pyroptosis represents a convergent pathogenic mechanism across diverse environmental insults.

#### 3.5.3 Integrated view: ferroptosis, pyroptosis, and the fibrotic network

When considered together with the ROS-driven oxidative stress (Section 3.1), TGF-β/Smad signaling (Section 3.2), and inflammatory cascades (Section 3.3), ferroptosis and pyroptosis emerge not as isolated pathways but as integral components of a self-reinforcing fibrotic network. Based on the evidence discussed above, we propose the following integrative working model: metal exposure → ROS overproduction → mitochondrial dysfunction and iron accumulation → ferroptosis of AECII cells → release of DAMPs → macrophage activation and NLRP3 inflammasome assembly → subsequent pyroptosis of additional epithelial and immune cells → sustained IL-1β/IL-18 release → fibroblast activation and collagen deposition. This interconnected cascade as summarized in **Figure 4**, suggests that targeting ferroptosis and/or pyroptosis may offer upstream intervention points that complement strategies directed at TGF-β/Smad or inflammatory hubs.

**Figure 4:**
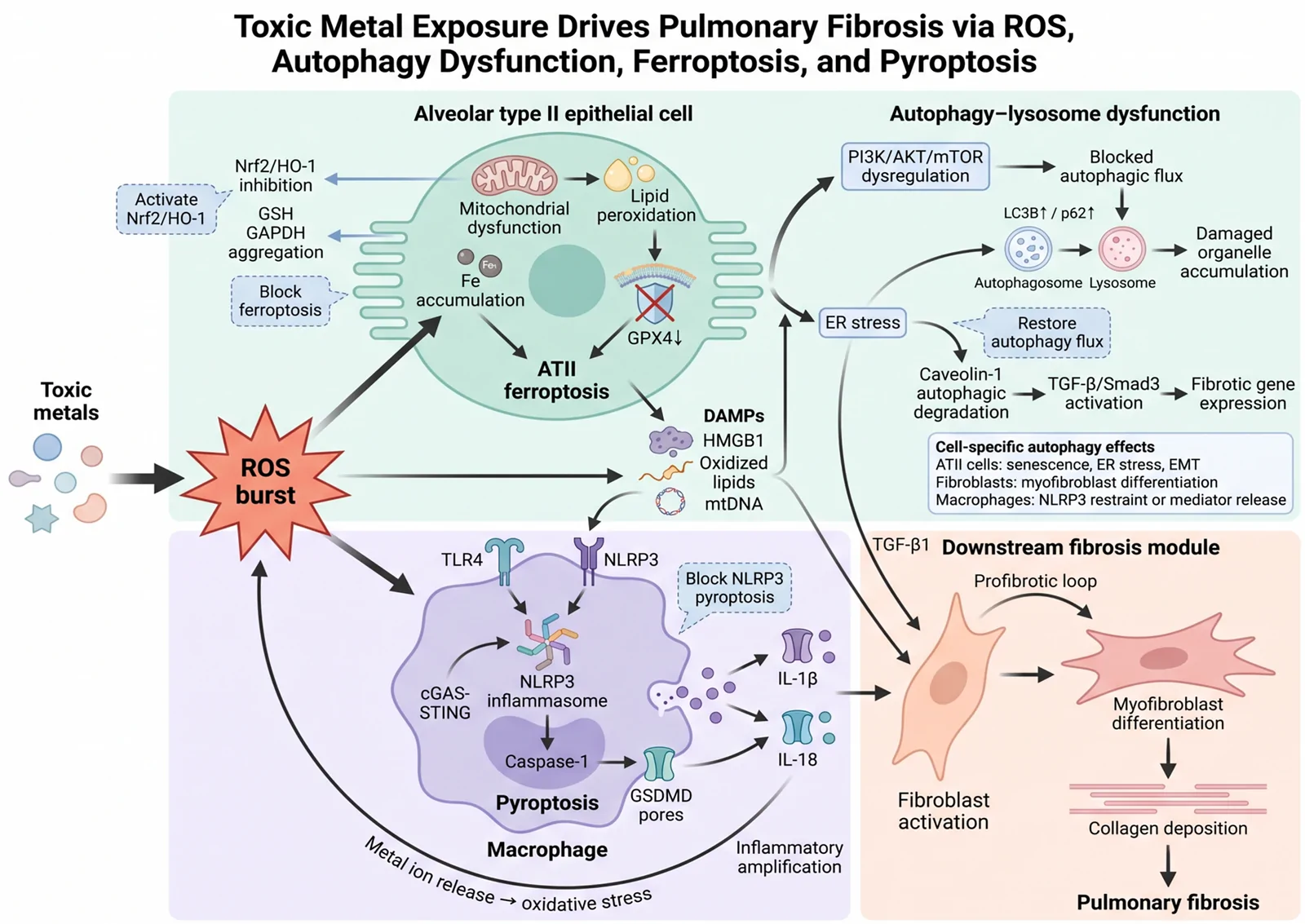
Cell-type-specific consequences of autophagy-lysosomal dysfunction in metal-induced pulmonary fibrosis. This image summarizes the dual and cell-type-dependent roles of autophagy in response to toxic metal exposure. Toxic metals disrupt autophagic flux primarily through the PI3K/AKT/mTOR pathway. The functional consequences of impaired autophagy vary markedly by cell type: in alveolar epithelial cells (particularly type II), autophagy inhibition promotes senescence, ER stress, and EMT, contributing to epithelial barrier dysfunction and fibroblast activation; in fibroblasts, suppressed autophagy permits excessive ECM production and myofibroblast differentiation; in macrophages, basal autophagy restricts NLRP3 inflammasome activation and M1 polarization, but excessive autophagy may promote the release of pro-fibrotic mediators that stimulate fibroblast activity. Mechanistically, ER stress-mediated caveolin-1 degradation relieves inhibition of TGF-β/Smad3 signaling, while autophagy activation via the Keap1/Nrf2 pathway alleviates oxidative stress and fibrosis. This cell-type specificity implies that global modulation of autophagy may produce opposing outcomes depending on the dominant cell type affected, highlighting the need for cell-targeted therapeutic strategies. AECII, alveolar epithelial type II cells; ECM, extracellular matrix; EMT, epithelial-mesenchymal transition; ER, endoplasmic reticulum; mTOR, mammalian target of rapamycin; Nrf2, nuclear factor erythroid 2-related factor 2; TGF-β, transforming growth factor-beta

Several unresolved questions merit future investigation. First, the temporal ordering of ferroptosis and pyroptosis during chronic metal exposure remains unclear—does one precede the other, or do they operate in parallel? Second, the cell-type specificity of these death pathways—whether AECII cells are uniquely susceptible compared to fibroblasts or macrophages—requires systematic mapping using single-cell approaches. Third, the relative contribution of ferroptosis versus pyroptosis to fibrotic outcomes across different metals is largely unknown. Fourth, the therapeutic potential of combined ferroptosis and pyroptosis inhibition, whether synergistic or redundant, has not been explored in metal-exposure models. Addressing these questions will be critical for translating insights from cell death biology into targeted anti-fibrotic interventions.

## 4 Targeted intervention strategies and emerging therapeutic approaches

Toxic metal exposure-induced pulmonary fibrosis involves complex molecular networks and remodeling of the cellular microenvironment. Targeted intervention strategies addressing its core pathogenic mechanisms are currently a major focus of research. From antioxidant and anti-inflammatory approaches to the regulation of classical fibrosis pathways, and on to emerging stem cell and nanodelivery technologies, multidimensional therapeutic explorations provide an important theoretical basis and clinical translation potential for blocking or reversing toxic metal-induced pulmonary toxicity. However, it is critical to emphasize that most of these strategies remain at the preclinical stage, with only a limited number having entered clinical trials, and none specifically approved for metal-induced pulmonary fibrosis. The following subsections systematically present the experimental evidence for each approach while explicitly indicating its current stage of clinical translation, so that readers may clearly distinguish between experimental efficacy and demonstrated clinical benefit.

### 4.1 Antioxidant and anti-inflammatory interventions targeting oxidative stress and inflammatory pathways

Oxidative stress and persistent inflammatory responses are the key initiating factors in toxic metal-induced pulmonary fibrosis. Toxic metals such as cadmium and molybdenum can target mitochondria, altering mitochondrial membrane permeability and inhibiting antioxidant enzyme activity, leading to excessive ROS production and accumulation of lipid peroxidation products such as 4-hydroxy-2-nonenal, which in turn trigger apoptosis and ferroptosis (6, 74, 75). In response to this mechanism, exogenous antioxidants and endogenous pathway modulators have demonstrated significant protective effects. For example, melatonin can alleviate cadmium-induced increases in ROS by activating the Nrf2/HO-1 signaling pathway, inhibit PERK-eIF2α-ATF4-mediated ER stress, and prevent iron overload and lipid peroxidation, thereby improving mitochondrial morphology and lung injury (35).N-acetylcysteine (NAC), a glutathione precursor, has shown efficacy in reducing oxidative damage in metal-exposed animal models and is widely used clinically as a mucolytic, though its specific anti-fibrotic efficacy in metal-induced fibrosis remains to be established in dedicated trials (68). In terms of inflammation regulation, toxic metal exposure activates the TLR4/NF-κB/NLRP3 inflammasome and cGAS-STING pathways, promoting M1 macrophage polarization and pro-inflammatory factor release (33, 72, 73). Inhibiting the cGAS-STING pathway or using specific extracts to modulate macrophage polarization can effectively reduce alveolar inflammation and block its progression to fibrosis (59, 72). Furthermore, modulating key products of oxidative stress and the relaxin signaling pathway is an effective strategy for reducing macrophage extracellular trap formation and lung tissue damage caused by mixed exposure (6, 30, 76). Regarding clinical development, most antioxidant and anti-inflammatory agents discussed here remain at the preclinical stage, with only a limited number having entered clinical use for other indications; dedicated trials for metal-induced pulmonary fibrosis are urgently needed.

Of the agents discussed above, N-acetylcysteine (NAC) is clinically available as a mucolytic and has been tested in various pulmonary conditions, but its specific efficacy in metal-induced pulmonary fibrosis has not been evaluated in dedicated trials. Melatonin has undergone clinical testing for other indications but remains at the preclinical stage for metal-induced lung fibrosis. Most other antioxidant and anti-inflammatory modulators discussed here—including Nrf2 activators and cGAS-STING inhibitors—are supported exclusively by *in vitro* or animal studies, with no clinical data available for fibrotic lung disease. Thus, while the preclinical rationale is strong, clinical translation remains a significant gap.

### 4.2 Targeted antifibrotic drugs that regulate TGF-β and autophagy pathways

Overexpression of the TGF-β/Smad signaling pathway and impaired autophagy play key roles in the progression of toxic metal-induced pulmonary fibrosis. Toxic metals such as cadmium and arsenic can directly bind to TGF-β receptors and activate the Smad pathway, or promote extracellular matrix deposition by inhibiting SIRT6, leading to phosphorylation of the AKT/PI3K signaling pathway (43, 48). Drugs targeting this pathway have shown considerable promise. Silymarin, a flavonoid compound, inactivates the PI3K/AKT/mTOR pathway by binding to Annexin A6, significantly inhibiting TGF-β1-induced fibroblast activation and myofibroblast differentiation (77). Salvia miltiorrhiza extract inhibits the Smad2/3 pathway and reduces collagen production (78). Clinically, the approved anti-fibrotic drugs pirfenidone and nintedanib—while not specifically developed for metal-induced fibrosis—have been shown to attenuate TGF-β signaling and are currently used in IPF management, suggesting potential repurposing opportunities for metal-related pulmonary fibrosis (9). At the same time, disruption of autophagy flux exacerbates alveolar epithelial cell senescence and EMT. Inhibiting autophagy with chloroquine or alleviating autophagy-lysosomal pathway-mediated degradation of lamin A/C can effectively reverse copper-induced cellular senescence and EMT (16, 79). Metformin, a widely used anti-diabetic drug, improves autophagy dysfunction and has entered clinical trials for anti-aging and fibrosis-related indications (80). SIRT6 inducers and flavonoids also alleviate the fibrotic phenotype by regulating autophagy and kinase pathways (16, 48, 80). Monitoring of CTHRC1, a downstream target of TGF-β, provides a potential biomarker for evaluating the efficacy of antifibrotic drugs such as pirfenidone (81). Currently, while pirfenidone and nintedanib are FDA-approved for IPF, most pathway-specific modulators (e.g., Smad7 agonists, SIRT6 inducers) remain in preclinical development, with Phase I/II trials ongoing for some mTOR and autophagy modulators in fibrotic diseases.

Pirfenidone and nintedanib are FDA-approved for idiopathic pulmonary fibrosis (IPF) and have well-established clinical safety and efficacy profiles in that context. However, their use in metal-induced pulmonary fibrosis represents an off-label repurposing opportunity rather than an approved indication, and no clinical trials have specifically evaluated their efficacy in metal-exposed populations. Metformin is a widely used anti-diabetic drug with an established safety profile and is currently being investigated in clinical trials for anti-aging and fibrosis-related indications, but its application to metal-induced fibrosis remains experimental. Silymarin, Salvia miltiorrhiza extract, SIRT6 inducers, and Smad7-targeted strategies are all at the preclinical stage, with no clinical data in fibrotic lung disease. Therefore, while repurposing of approved drugs offers a faster translational pathway, dedicated clinical evaluation in metal-induced fibrosis cohorts is urgently needed.

### 4.3 Toxic metal chelators and adjuvant therapies to promote the elimination of toxic metals from the lungs

Promoting the excretion of toxic metals and restoring metal ion homeostasis are direct strategies for treating toxic metal-induced pulmonary toxicity. Imbalances in trace elements such as copper, zinc, and iron in the lungs can disrupt redox homeostasis and drive fibrosis progression; therefore, restoring the balance of this “metal triad” holds significant therapeutic potential (7, 8). Copper chelators have demonstrated significant efficacy in anti-fibrosis therapy. Tetrathiomolybdate significantly improves bleomycin-induced pulmonary fibrosis and lung compliance by downregulating vascular endothelial growth factor (VEGF) expression, reducing vascular density, and decreasing collagen deposition (82). Another copper chelator, trientine (triethylene tetramine), inhibits copper transporter 1 and lysine oxidase expression, upregulates Nrf2, and reduces TNF-α and IL-6 levels, thereby reversing pulmonary structural damage (83). However, the clinical application of copper chelators faces important challenges: tetrathiomolybdate can induce dose-dependent myelosuppression and hepatotoxicity, while trientine may cause gastrointestinal intolerance and iron deficiency anemia due to off-target metal binding. Furthermore, because copper is an essential cofactor for superoxide dismutase and cytochrome c oxidase, excessive chelation risks impairing vital antioxidant and mitochondrial functions, necessitating careful dose titration and biomarker monitoring in any clinical application. Regarding the cadmium-induced ferroptosis mechanism, the ferroptosis inhibitor Liproxstatin-1 effectively alleviates glycolytic dysfunction caused by glyceraldehyde-3-phosphate isomerase 1 aggregation, reducing inflammatory and fibrotic gene expression (11). Iron chelators and zinc supplementation have also shown preclinical efficacy in restoring metal homeostasis and limiting oxidative damage, though clinical data remain sparse (7, 8). Furthermore, specific microorganisms, such as Bacillus species, and their bioadsorption and efflux mechanisms provide a molecular-level approach to intervening in toxic metal detoxification and clearance in both environmental and bodily contexts, though this strategy remains largely exploratory (84).Overall, chelation-based therapies for pulmonary fibrosis are currently at the preclinical to early Phase I/II stage, with no metal chelator specifically approved for fibrotic indications.

Tetrathiomolybdate and trientine are clinically available for Wilson’s disease (copper overload disorder) and have established safety profiles in that context. Their evaluation in pulmonary fibrosis, however, remains at the preclinical stage, with all published evidence derived from bleomycin-induced fibrosis models rather than metal-exposed animals or patients. Liproxstatin-1 and other ferroptosis inhibitors are experimental compounds with no clinical use in any indication. Iron chelators and zinc supplementation have clinical applications in other conditions, but their efficacy in metal-induced pulmonary fibrosis has not been tested in humans. Overall, despite the strong mechanistic rationale, no chelation-based therapy has advanced to clinical trials specifically for metal-induced pulmonary fibrosis, representing a major translational gap.

### 4.4 Exploring emerging therapies: stem cell therapy and nanomedicine delivery systems

Advances in biomaterials and regenerative medicine have opened new avenues for targeted interventions in toxic metal-induced pulmonary fibrosis. Mesenchymal stem cell (MSC) therapy has demonstrated significant preclinical potential. Umbilical cord-derived MSCs and adipose-derived MSCs have been shown to restore metal-ion homeostasis in the lungs, modulate the immune microenvironment (promoting M2-to-M1 shift in appropriate contexts), and alleviate fibrotic remodeling through paracrine mechanisms involving exosomes and growth factors (7, 85). A representative study by Perrot et al. demonstrated that MSC-derived exosomes carrying anti-fibrotic microRNAs can effectively reduce collagen deposition in bleomycin-induced fibrosis models (85). However, challenges including low engraftment rates, potential tumorigenicity, and batch-to-batch variability remain significant barriers to clinical translation, with most MSC therapies for pulmonary fibrosis currently in Phase I/II trials (85).

In the areas of drug delivery and tissue engineering, nanomaterial-based inhalation systems can enhance targeted accumulation and bioavailability of drugs in the lungs. For example, polyethylene glycol (PEG)-coated liposomes and poly(lactic-co-glycolic acid) (PLGA) nanoparticles loaded with anti-fibrotic agents have shown improved lung retention and reduced systemic toxicity in preclinical models (30). When combined with exosome therapy, nano-formulations can synergistically enhance antioxidant and anti-inflammatory effects (30, 85). Furthermore, studies using photopolymerizable hydrogels to mimic the physical properties of healthy and fibrotic lung ECM have found that hyaluronic acid-containing matrices can effectively buffer fibrosis-related gene expression induced by cyclic mechanical strain. This provides a theoretical basis for developing implantable or inhalable biomaterials with mechanotransduction-regulating functions (86). Despite promising preclinical progress, the clinical adoption of nanomedicine for pulmonary fibrosis faces hurdles including long-term biocompatibility, manufacturing scalability, and regulatory approval pathways, with most candidates still in preclinical or early Phase I evaluation (85).

Mesenchymal stem cell (MSC) therapy has entered clinical trials for IPF and other fibrotic diseases, but its application to metal-induced pulmonary fibrosis has not been specifically investigated in any clinical study. Significant barriers—including low engraftment rates, batch-to-batch variability, potential tumorigenicity, and uncertain dosing regimens—remain unresolved even for IPF, and these challenges are magnified for metal-induced fibrosis where concurrent metal toxicity may affect MSC survival and function. Nanomedicine delivery systems are largely at the preclinical optimization stage, with no clinical data available for pulmonary fibrosis of any etiology. Photopolymerizable hydrogels and mechanotransduction-modulating biomaterials remain at the proof-of-concept stage *in vitro*.

However, the translational potential of nanomedicines and EV-based therapies for metal-induced pulmonary fibrosis is severely constrained by lung-specific physical delivery barriers that are often overlooked in preclinical proof-of-concept studies. First, the dense and extensively crosslinked extracellular matrix (ECM) characteristic of fibrotic lungs—enriched in collagen I, fibronectin, and hyaluronan—functions as a physical barrier that restricts nanoparticle penetration and diffusion (53, 86). Second, activated alveolar macrophages in the fibrotic microenvironment efficiently recognize and clear synthetic nanoparticles and EVs through phagocytic and scavenger receptor-mediated pathways, substantially reducing their bioavailability and target-site accumulation (85). These two barriers operate synergistically: ECM entrapment prolongs particle residence in perivascular spaces, increasing the opportunity for macrophage recognition and clearance, while the inflammatory milieu further upregulates macrophage scavenger receptor expression, creating a self-reinforcing clearance loop.

To overcome these obstacles, several engineering strategies have been explored. For ECM penetration, smaller particle sizes (≤100 nm) and surface modifications with ECM-degrading enzymes have been shown to enhance diffusion through crosslinked matrices (86). For macrophage evasion, surface PEGylation, CD47 “don’t eat me” peptide coating, or encapsulation in red blood cell-derived membranes can reduce non-specific uptake by phagocytes (85). More recent approaches employ macrophage-depleting agents as a pre-treatment step to transiently reduce the clearance burden before nanoparticle administration, though this strategy carries infection risks that require careful management. Additionally, extracellular vesicle engineering, including the incorporation of targeting ligands or fusogenic proteins that facilitate endosomal escape—can improve both tissue homing and intracellular delivery of therapeutic cargoes (30, 85). However, most of these engineering solutions have been validated primarily in non-fibrotic or acute injury models. Their efficacy in the uniquely stiff, inflamed, and macrophage-rich microenvironment of chronic metal-induced fibrosis remains largely unexplored and warrants dedicated investigation. Furthermore, the potential synergistic application of ECM-normalizing agents combined with nanoparticle delivery has not been tested in metal-exposure models, representing a promising but untapped direction for future research. Thus, while these emerging approaches hold considerable theoretical promise, they are the farthest from clinical translation among all strategies discussed in this section.

### 4.5 Combination therapy: targeting the fibrotic network synergistically

Given the interconnected nature of the molecular networks driving metal-induced pulmonary fibrosis, single-agent therapy is unlikely to achieve optimal efficacy. The extensive crosstalk among ROS production, TGF-β/Smad signaling, inflammation, and impaired autophagy provides a strong rationale for combination approaches. For instance, combining an Nrf2 activator (e.g., sulforaphane or melatonin) with a TGF-β receptor inhibitor (e.g., a small-molecule ALK5 inhibitor) could simultaneously reduce the oxidative trigger and the central fibrotic driver, potentially yielding synergistic effects greater than either agent alone (35, 43). Similarly, co-administration of a copper chelator with an autophagy enhancer (e.g., rapamycin or metformin) addresses both the metal-specific toxicity and the cellular clearance defect that perpetuates fibrosis (16, 43, 80). Preclinical evidence supports this concept: in cadmium-induced fibrosis models, combining N-acetylcysteine with the ferroptosis inhibitor Liproxstatin-1 showed greater reduction in fibrotic markers than either treatment alone (11, 66). In copper-induced EMT models, the concurrent use of trientine and chloroquine synergistically reversed cellular senescence and ECM deposition (16, 79). However, the optimal timing, dosing, and sequence of combination therapies remain poorly understood, and potential drug-drug interactions or overlapping toxicities require systematic evaluation. Future research should leverage network pharmacology and systems biology approaches to identify rational drug combinations and personalized regimens based on individual exposure profiles and genetic susceptibility. This integrated, multi-target strategy represents a paradigm shift from the current single-agent approach and holds considerable promise for improving outcomes in toxic metal-related pulmonary fibrosis.

The combination therapy strategies proposed here are based on preclinical evidence and theoretical network pharmacology considerations rather than clinical data. No combination regimen has been tested in animal models of metal-induced fibrosis, let alone in human trials. The extensive crosstalk among pathways provides a strong mechanistic rationale, but the optimal drug combinations, dosing schedules, and timing of administration remain entirely undefined. Furthermore, potential drug-drug interactions and overlapping toxicities—particularly relevant when combining agents with narrow therapeutic windows (e.g., chelators) or immunomodulatory effects—require systematic preclinical evaluation before any clinical consideration. Thus, combination therapy represents a future direction rather than an immediately translatable strategy, and its clinical feasibility remains to be established.

## 5 Limitations, controversies, and unresolved issues in current research

Although significant progress has been made in research on toxic metals causing pulmonary fibrosis, substantial limitations and controversies remain at multiple levels. Animal models struggle to simulate the chronic, low-dose, mixed-exposure patterns observed in real-world populations, and most current protocols model acute metal toxicity rather than the insidious, progressive fibrotic remodeling that characterizes human disease. The dose-response relationship for mixed metals is nonlinear and influenced by metal interactions, making it difficult to determine safe thresholds. Existing mechanistic studies have largely focused on the activation of single signaling pathways, lacking an in-depth understanding of feedback regulation and network-level compensatory mechanisms. These shortcomings severely limit causal inference and clinical translation. There is an urgent need to develop research models that more closely reflect real-world exposure scenarios, integrating multi-omics with prospective cohorts to advance precision prevention and treatment strategies.

### 5.1 Differences between animal models and real-world human exposure scenarios

There remains a significant translational gap between current research in animal models and real-world human exposure scenarios—a gap that extends beyond differences in exposure routes and doses to encompass fundamental differences in pathobiological progression. Most animal studies employ short-term, high-dose, or single-route exposure methods. Examples include short-term exposure via intratracheal injection of cadmium chloride; combined exposure to specific concentrations of nickel, copper, and arsenic via inhalation; and 50-day exposure to molybdenum and cadmium (87–89). However, real-world human exposure typically involves long-term, low-dose, and complex, mixed scenarios across multiple routes. Examples include long-term occupational exposure to multiple toxic metals in metal casting, cement production, or welding, as well as chronic mixed exposure in the general population from environmental sources (19, 23, 90, 91). A critical distinction that is often blurred in the literature—and one that carries substantial pathophysiological and translational implications—is the difference between acute metal-induced lung injury and chronic metal-driven progressive fibrosis. Most current animal models utilize short-term, high-dose exposure protocols that produce severe oxidative stress, widespread epithelial necrosis, and robust acute inflammation within days to weeks. While these models effectively recapitulate the early events of acute lung injury, they do not necessarily replicate the insidious, slowly progressive fibrotic remodeling that characterizes human pulmonary fibrosis following long-term, low-level occupational or environmental metal exposure. In the human context, metals accumulate in lung tissues over years or decades, driving persistent low-grade inflammation, recurrent cycles of epithelial micro-injury and repair, gradual fibroblast activation, and progressive extracellular matrix deposition—often in the absence of overt acute episodes. This chronic accumulation-driven process involves distinct molecular and cellular dynamics, including adaptive metabolic reprogramming, epigenetic remodeling, and progressive loss of tissue regenerative capacity, which may not be adequately captured by acute high-dose models. Therefore, findings derived from acute instillation models should be interpreted with caution when extrapolating to the pathogenesis of chronic fibrotic disease, and the field would benefit from a clearer distinction between metal-induced acute lung toxicity and metal-driven chronic fibrogenesis as mechanistically and clinically distinct entities. Some studies have attempted to simulate human occupational exposure levels by administering 6-month systemic exposures to lead and cadmium (92).Most animal models still struggle to accurately reflect the complex metabolic kinetics, long-term accumulation effects, and multi-organ interactions observed in humans. Furthermore, while *in vitro* cellular experiments can reveal the direct effects of specific metals, such as manganese and lead, on pulmonary fibroblasts and alveolar epithelial cells, they lack the complexity of the *in vivo* microenvironment, posing significant challenges to the translation of basic research findings into clinical prevention and treatment strategies (50).

To bridge this gap, future animal model designs should incorporate several key refinements. First, exposure protocols should adopt intermittent or fluctuating concentration regimes rather than constant high-dose administration, better reflecting the episodic nature of occupational and environmental exposure. Second, multi-route administration should be considered to mimic the integrated human exposure pathway. Third, the use of aged animals or those with pre-existing comorbidities would better represent the susceptible human populations most at risk of metal-induced fibrosis. Fourth, extended exposure durations (≥6 months) with post-exposure recovery periods are necessary to capture the chronic accumulation and progressive nature of fibrotic remodeling.

Among these refinements, the development and validation of chronic low-dose inhalation models deserve particular priority. Such models—employing aerosolized metal compounds at environmentally or occupationally relevant concentrations over 6–12 months with longitudinal monitoring of lung function, tissue pathology, and molecular biomarkers—would far more faithfully recapitulate the human exposure scenario than acute instillation. Importantly, these chronic models should incorporate recovery periods to assess the reversibility of early fibrotic changes and identify the irreversibility threshold—the point at which early injury transitions to self-perpetuating fibrosis. Additionally, the use of real-time *in vivo* imaging and longitudinal bronchoalveolar lavage in such models would enable the identification of dynamic biomarker signatures that track disease progression, offering valuable translational tools for early diagnosis and therapeutic monitoring. While resource-intensive, the establishment of standardized chronic low-dose inhalation models across multiple metal types is an urgent unmet need in the field and a prerequisite for credible preclinical evaluation of therapeutic interventions. Such refined models, while more resource-intensive, would substantially enhance the translational relevance of preclinical findings and provide more reliable platforms for therapeutic evaluation.

### 5.2 Controversies surrounding dose-response relationships for mixed toxic metal exposure and the threshold problem

The dose-response relationship for mixed toxic metal exposure is extremely complex and often exhibits nonlinear characteristics, leading to significant controversy regarding the definition of safe thresholds. Epidemiological studies have shown nonlinear dose-response relationships between toxic metal exposure and various health outcomes: mercury exhibits an inverted U-shaped relationship with the lung function index FEV_1_/FVC (22). Lead, cadmium, and manganese are nonlinearly associated with accelerated phenotypic aging and exhibit threshold effects (21). Cadmium also exhibits a nonlinear relationship with forced vital capacity (19). Furthermore, interactions among metals complicate the determination of thresholds—arsenic exhibits negative interactions with other metals in certain models, whereas manganese exhibits positive interactions with nickel (93). *In vitro* experiments have also confirmed that cadmium and copper exhibit the strongest synergistic effects, whereas iron and zinc exhibit the strongest antagonistic effects (28). Currently, many studies fail to report clear dose-response thresholds, resulting in a lack of precise quantitative evidence to inform environmental standards and clinical risk assessments (21, 29).

To address the complexity of mixed-metal exposure, advanced statistical methods have been increasingly applied in environmental epidemiology. Methods such as Bayesian Kernel Machine Regression (BKMR) and Weighted Quantile Sum (WQS) regression allow for the simultaneous evaluation of multiple correlated exposures, identification of important mixture components, and characterization of nonlinearity and interactive effects without assuming linearity or independence (91, 94). BKMR can estimate the joint effect of a metal mixture and identify potential interactions through a flexible exposure-response function, while WQS regression provides a single weighted index of mixture exposure. However, the application of these sophisticated methods to pulmonary fibrosis outcomes remains surprisingly limited. Most existing studies have focused on renal function, neurodevelopment, or cardiovascular endpoints, leaving the metal mixture-fibrosis association largely unexplored using these advanced analytical frameworks. Future epidemiological investigations should prioritize the application of BKMR, WQS, and similar approaches to characterize the combined effects of toxic metal mixtures on fibrotic lung disease, which would provide more robust evidence for revising environmental guidelines and identifying high-risk exposure profiles.

### 5.3 Insufficient analysis of feedback regulation and compensatory mechanisms in molecular mechanism networks

Current research on the mechanisms underlying toxic metal-induced pulmonary fibrosis has largely focused on the linear activation of single signaling pathways, such as excessive activation of the TGF-β/Smad signaling pathway, oxidative stress and ferroptosis cascades, and inflammasome activation. However, there remains a lack of understanding of the complex feedback regulation and compensatory mechanisms within the molecular network (33, 43, 88). For example, trace element imbalances triggered by toxic metal exposure involve complex interactions and compensatory regulation, but the dynamic feedback processes underlying their role in remodeling the pulmonary microenvironment have not yet been fully elucidated (7, 8). Intercellular paracrine feedback mechanisms—such as lead-treated lung fibroblasts secreting conditioned medium to upregulate fibrosis and apoptosis markers in alveolar epithelial cells and activate the ERK signaling pathway—remain poorly characterized at the molecular level (50).Compensatory regulatory networks following an imbalance in M1/M2 macrophage polarization within the immune microenvironment remain underexplored (50). Furthermore, epigenetic feedback regulations, such as the role of the miRNA sponge model in toxic metal exposure and obstructive lung dysfunction, require in-depth analysis, as does the impact of nickel exposure on chromatin structure and cellular regulatory processes (20, 95).

To illustrate the importance of feedback regulation with a concrete example: TGF-induced microRNA-21 (miR-21) has been shown to directly target and suppress Smad7, the endogenous inhibitory Smad, thereby creating a positive feedback loop that amplifies TGF-β signaling and promotes fibrosis progression (96). This mechanism has been partially characterized in various fibrotic conditions. However, the compensatory regulatory responses that might counteract this feed-forward loop remain poorly understood—for instance, whether prolonged miR-21-mediated Smad7 suppression triggers the compensatory upregulation of other inhibitory Smads, or whether protein phosphatases that dephosphorylate Smad3 are concurrently modulated to buffer the hyperactive signaling. Similarly, while chronic metal exposure is known to suppress SIRT6 expression and enhance AKT/PI3K signaling (48), the feedback mechanisms that might restore SIRT6 levels or activate parallel antioxidant defenses remain largely unexplored. These knowledge gaps are not merely academic; they have direct therapeutic implications. A deeper understanding of feedback and compensatory circuits could identify novel intervention points and predict resistance mechanisms to current anti-fibrotic agents, ultimately guiding the development of more durable and effective combination therapies. Addressing these gaps will require integrated approaches combining time-series omics profiling, computational network modeling, and functional validation in physiologically relevant models.

## 6 Future research directions and prospects

Given the limitations and unresolved issues identified above, future research in toxic metal-induced pulmonary fibrosis should prioritize the following specific scientific questions. Addressing these questions will require integrated approaches combining advanced toxicological models, multi-omics technologies, and rigorous epidemiological designs.

### 6.1 What are the quantitative threshold interactions among arsenic, cadmium, and lead in promoting human lung fibroblast activation?

While epidemiological studies have established dose-response relationships for individual metals (17–19), the interactive effects of mixed metal exposure—whether synergistic, additive, or antagonistic—remain poorly quantified at the cellular level. Current *in vitro* studies have largely focused on single metals or simple binary combinations, yet real-world exposure involves complex mixtures with variable ratios (28, 91). Future research should systematically map the response surface of human lung fibroblasts to physiologically relevant concentration matrices of As, Cd, and Pb, using high-content imaging and transcriptomic readouts to define interactive thresholds. Such data would directly inform mixture risk assessment models and provide a mechanistic basis for interpreting epidemiological findings. Advanced statistical designs such as response surface methodology (RSM) or fractional factorial designs could efficiently model these multi-dimensional interactions.

### 6.2 Can single-cell and spatial transcriptomics identify metal-specific fibroblast subpopulations and their spatial organization in fibrotic lungs?

Bulk tissue transcriptomics has revealed global gene expression changes in metal-induced fibrosis, but these analyses average out the functional heterogeneity of fibroblasts—a cell type now recognized to encompass multiple subpopulations with distinct pro-fibrotic, pro-inflammatory, and matrix-remodeling phenotypes (85). Single-cell RNA sequencing (scRNA-seq) and spatial transcriptomics offer unprecedented opportunities to dissect whether specific toxic metals drive the emergence of unique fibroblast subsets or converge on common fibrogenic programs. For example, does cadmium preferentially expand a particular myofibroblast cluster expressing distinct ECM genes compared to nickel? Do metal-exposed fibroblasts exhibit unique ligand-receptor interactions with adjacent epithelial or immune cells that are not observed in idiopathic fibrosis? Answering these questions would not only refine our mechanistic understanding but also identify cell type-specific therapeutic targets and potential biomarkers for early detection.

### 6.3 What is the temporal sequence of ROS production, inflammatory activation, and TGF-β signaling during chronic metal exposure *in vivo*?

Although the individual contributions of oxidative stress, inflammation, and TGF-β signaling to metal-induced fibrosis are well established, the temporal dynamics and causal ordering of these events remain unclear. Do persistent low-level metals first trigger low-grade ROS production that slowly amplifies into overt inflammatory responses, or do metal-induced epithelial injury directly activate TGF-β signaling independently of inflammation? At what time point does the process transition from reversible injury to irreversible fibrosis? Addressing these questions requires time-series experimental designs with frequent, longitudinal sampling of bronchoalveolar lavage fluid, lung tissue, and circulating biomarkers in well-controlled animal models. Integration with mathematical modeling of signaling dynamics could help predict critical intervention windows and identify biomarkers that signal the transition from early injury to established fibrosis.

### 6.4 Can multi-target combination therapies be rationally designed based on network pharmacology to overcome feedback and compensatory mechanisms?

As discussed in Section 4.5, combination therapy holds considerable promise, but current efforts remain largely empirical (11, 16, 35). The incomplete understanding of feedback loops and compensatory responses—such as the miR-21/Smad7 positive feedback loop and the poorly characterized compensatory upregulation of other inhibitory Smads or phosphatases—represents a major barrier to rational combination design. Future research should employ network pharmacology and systems biology approaches to map the regulatory network of metal-induced fibrosis, identifying critical nodes and edge redundancies that confer resistance to single-agent therapy. Computational simulations of network perturbations could predict synergistic drug combinations and optimal dosing schedules, which can then be validated in organoid or animal models. Additionally, longitudinal monitoring of pathway activity in response to therapy could reveal adaptive resistance mechanisms in real time, enabling iterative refinement of combination regimens.

### 6.5 What are the predictive values of exposomic and multi-omic biomarkers for early detection and personalized risk stratification?

The ultimate translational goal is to identify individuals at highest risk of metal-induced fibrosis before irreversible lung damage occurs. While traditional epidemiological studies have identified associations between metal exposure and fibrosis, individual susceptibility varies considerably due to genetic polymorphisms, epigenetic modifications, and lifestyle factors (10, 21, 22). Future large-scale prospective cohort studies should integrate comprehensive exposomic profiling, genetic and epigenetic screening, and advanced lung imaging to develop personalized risk prediction models. Machine learning approaches could integrate multi-dimensional data to identify predictive biomarker panels and establish clinically actionable risk scores. Importantly, such cohorts should include diverse populations across different geographic regions and occupational settings to ensure generalizability and address the racial/ethnic susceptibility differences suggested by early hotspot observations (10).

## 7 Conclusion

This review establishes toxic metal exposure as a significant environmental risk factor for pulmonary fibrosis, with consistent epidemiological evidence across diverse populations and dose-response patterns for arsenic, lead, and copper. Mechanistically, we have moved beyond a linear, single-pathway view to reveal a complex, interconnected signaling network. In this network, ROS overproduction serves as the initiating trigger and TGF-β/Smad functions as the central hub—both of which are supported by consistent and reproducible evidence. Inflammatory, autophagic, and epigenetic dysregulation—along with non-apoptotic cell death pathways including ferroptosis and pyroptosis—act as interdependent amplifying loops. However, the evidence for these loops varies considerably, with some interactions being comparatively well-characterized, whereas others remain largely unexplored and require further experimental validation. A comparative summary of metal-specific versus shared mechanisms is provided in **Table 1**, which serves as a quick reference for readers to assess the evidence base for each metal-pathway relationship.

This network perspective represents a paradigm shift with profound implications. Rather than targeting individual pathways in isolation, future therapeutic strategies must embrace the reality of pathway crosstalk, positive feedback, and compensatory adaptation. The emerging recognition that macrophages, fibroblasts, and epithelial cells each respond differently to autophagy modulation—and that metal mixtures produce nonlinear, interactive effects—further underscores the inadequacy of reductionist approaches.

While the therapeutic strategies summarized in Section 4 offer multiple mechanistic entry points for intervention, it is imperative to recognize that the overwhelming majority are supported exclusively by preclinical evidence. Only pirfenidone and nintedanib are FDA-approved for pulmonary fibrosis, and their application to metal-induced fibrosis remains off-label and unevaluated. No antioxidant, autophagy modulator, metal chelator, stem cell therapy, or nanomedicine formulation has been tested in clinical trials specifically for metal-induced pulmonary fibrosis. This translational gap—from robust mechanistic rationale to clinical proof-of-concept—represents the single most important challenge for the field and should temper expectations while motivating rigorous translational research.

The next frontier lies at the intersection of network pharmacology and real-world exposomics: integrating multi-omics profiling, single-cell resolution, and advanced mixture modeling to design multi-target interventions that simultaneously address oxidative triggers, fibrotic drivers, and feedback resistance mechanisms. Such an integrated framework, bridging environmental toxicology with precision medicine, holds the key to achieving the ultimate goals of early warning, accurate risk stratification, and effective combination therapy for toxic metal-related pulmonary fibrosis.

## Glossary

4-HNE: 4-hydroxy-2-nonenal
AECII: alveolar epithelial type II cells
BKMR: Bayesian Kernel Machine Regression
cGAS: cyclic GMP-AMP synthase
COPD: chronic obstructive pulmonary disease
CTGF: connective tissue growth factor
CTHRC1: collagen triple helix repeat containing 1
DAMP: damage-associated molecular pattern
ECM: extracellular matrix
EMT: epithelial-mesenchymal transition
ER: endoplasmic reticulum
EV: extracellular vesicle
FEV₁: forced expiratory volume in 1 second
FVC: forced vital capacity
GAPDH: glyceraldehyde-3-phosphate isomerase 1
GPX4: glutathione peroxidase 4
GSDMD: gasdermin D
HMGB1: high-mobility group box 1
IL: interleukin
IPF: idiopathic pulmonary fibrosis
JAK: Janus kinase
MAPK: mitogen-activated protein kinase
MMP: matrix metalloproteinase
MSC: mesenchymal stem cell
mTORC1: mammalian target of rapamycin complex 1
NAC: N-acetylcysteine
NF-κB: nuclear factor kappa-B
NLRP3: NOD-like receptor family pyrin domain containing 3
Nrf2: nuclear factor erythroid 2-related factor 2
PEF: peak expiratory flow
PEG: polyethylene glycol
PI3K: phosphatidylinositol 3-kinase
PLGA: poly(lactic-co-glycolic acid)
PM: particulate matter
ROCK: Rho-associated protein kinase
ROS: reactive oxygen species
scRNA-seq: single-cell RNA sequencing
SIRT6: sirtuin 6
STAT: signal transducer and activator of transcription
STING: stimulator of interferon genes
TGF-β: transforming growth factor-beta
TLR4: Toll-like receptor 4
TNF-α: tumor necrosis factor-alpha
VEGF: vascular endothelial growth factor
WQS: Weighted Quantile Sum.
